# Diseases of the Eye

**Published:** 1900-08

**Authors:** 


DISEASES OF THE EYE. By Edward Nettleship, F.R.C.S., Surgeon to Royal London Ophthalmic Hospital. Revised and edited by Wm. Campbell Posey, A.B., M.D., Assistant Surgeon Wills' Eye Hospital. Philadelphia. Sixth American edition. Published by Lea Brothers & Co., Philadelphia.

Few works have been so popular as a text-book as the above. The main reasons are two: first, because it is thorough, explicit and eminently practical; second, because it is not an expensive book for the medical student to purchase. In this last edition the work has been thoroughly revised, which makes it still hold the place of a standard edition. The illustrations are good, and it must still hold its place as a popular text-book. R.

